# Practice of Postoperative Pain Management in Under-Five Children in A Tertiary Hospital: A Prospective Crossectional Study

**DOI:** 10.4314/ejhs.v32i6.8

**Published:** 2022-11

**Authors:** Belachew Dejene Wondemagegnehu, Mekdelawit Mesfin Tadess

**Affiliations:** 1,2 Addis Ababa University, College of Health Sciences, Department of Surgery, Division of Paediatric Surgery, Ethiopia, Addis Ababa

**Keywords:** Pain, postoperative pain, under-five children, pain assessment tool

## Abstract

**Background:**

Despite advancements in pain management, children have remained undertreated for postoperative pain. Data regarding the practice of post-operative pain management in paediatric patients remains less available in the developing world. This study was aimed at evaluating practice of postoperative pain management in under five children.

**Methods:**

A prospective cross sectional one-year study was conducted on all paediatric patients who underwent major paediatric surgical procedures from February 1, 2020 to January31, 2021, at a tertiary hospital in Addis Ababa, Ethiopia.

**Result:**

A single type of analgesic medication was used in 67.1% patients. Analgesic medications were administered on standing base only in 64.4% of patients. Patients' charts had no documentation of pain assessment both in the neonatal intensive care units and wards.32.89 % of assessed patients had moderate to severe pain record.

**Conclusion:**

Significant number of patients suffer from postoperative pain because of absence of proper pain assessment and inadequate administration of analgesic medication.

## Introduction

Although every living organism is familiar with pain, it is a complex and multidimensional experience which is hard to clearly define specially in children (1–3) Cheng, Foster, and Hester 2003 and Cheng, Foster, and Huang 2003 suggest that it is a distressful and an unpleasant feeling which when left unrelieved, could affect the quality of life of an individual (4–6).

The importance of adequate pain therapy is unquestionable. Although the Declaration of Montreal (September 2010) states that “Access to Pain Management is a Fundamental Right”, insufficient pain management remains to be a problem in 80% of population in more than 150 countries. Majority of victims of inadequate pain management are the elderly, pregnant, and breastfeeding women, children, drug addicted persons, and the mentally ill. Despite efforts to advance perioperative pain management of children, there are still a substantial number of children suffering from perioperative pain (7). Inflammatory reactions due to surgical trauma causes acute pain in post-operative period. Pain is a complex phenomenon that include behavioral, developmental, emotional, physiological, and sociocultural components. The emotional component is particularly strong in children. As post-operative pain has significant effect in children's healing and recovery process, professionals should give emphasis in preventing and managing postoperative pain (8).

Pain Management education in medical and paramedical schools in Ethiopia is poor. Absence of adequate studies and standard guidelines to pain management could be mentioned as the main reasons for poor pain management practice in the country (9).

There was no study done on practice of postoperative pain management in under five children in Ethiopia, therefore, this study could be a stepping stone for further studies and could help to develop applicable pain management guidelines.

## Methods and Materials

**Study area and design:** A hospital based prospective cross-sectional study was conducted on a total of 235 paediatric post-operative patients operated from February 1,2020 to January 31, 2021 at Tikur Anbesa Specialized Teaching Hospital; Addis Ababa, Ethiopia, where full time paediatric surgical service is given by the paediatric surgical residents, fellows and consultants.

**Sampling and study population**: The sample size was determined using the formula for single population proportion by assuming 5% marginal error and 95% confidence interval (α (alpha)=0.05). The proportion (p) = 50% was taken as there was no research done in the same setting concerning pattern of postoperative pain management in under five children


$$\begin{array}{ccccc} \text{n=} & \frac{{(\text{z)}}^{\text{2}}\text{p(1-p)}}{{\text{d}}^{\text{2}}} & = & \frac{{\left(1.96\right)}^{2}\text{ x 0.5(1-0.5)}}{{\left(0.05\right)}^{2}} & =\;384 \end{array}$$

n = the required sample size, z = the value of the standard normal curve score corresponding to the given confidence by taking additional 20% contingency for non-response rate, the sample size is: 196 +20 % (non-response rate) = 196+39= 235 All patients who fulfilled inclusion criteria were included in the study.

**Inclusion criteria**: All Paediatric patients under the age of 5 years that underwent major paediatric surgical procedures at Tikur Anbesa specialized Hospital in their first and second postoperative days during the study period were included and all selected cases responded to the questions.

**Exclusion criteria**: Incomplete documentations and use of wrong type of assessment tool.

**Data management and interpretation**: Data was collected using structured questionnaire and patients' post-operative pain was assessed using a standardized questionnaire with pain assessment tools: Neonatal /Infant pain assessment tools (NIPS) was used for neonates and infants, FLACC scale (Face, Leg, Activity, Cry and Consolability) for those between the age of 1year and 5 years and Collected data was analyzed using SPSS version 23, only 10 patients were excluded from analysis for gross incompleteness and use of wrong type of assessment tool.

Interpretation of Mild, Moderate and Severe pain was made based on the standard values of the tools. ([NIPS: 0–2 = no pain to mild pain, 3–4 = moderate pain, >4 = severe pain.], [And for FLACC scale: Relaxed and comfortable=0, Mild discomfort=1–3, Moderate pain=4–6, Severe discomfort or pain or both=7–10]).

**Ethical considerations**: All methods were carried out in accordance with the ethical standards as laid down in 1964 Declaration of Helsinki and its later amendments for which reason, Ethical clearance was obtained from institutional review board of Addis Ababa University, College of Health Sciences School of Medicine and an informed consent was taken from parents /legal guardians of each patient before data collection.

## Results

A total of 235 paediatric post-operative patients were assessed for post-operative pain using a standardized questionnaire with pain assessment tool of which half (50.7%) patients were between the age of 1 year and 5 years. Infants and neonates account for 31.1% and 18.2% of the cases respectively. 57.3% of the cases were male patients making male to female ratio 1.34:1 of these 39(17.3%) were admitted to the neonatal Intensive care unit (NICU) and 186 (82.6%) to the wards. The types of surgeries conducted during the study period were: 26.6% laparotomies, 14.6% stoma reversals, 14.6% epispadias/ hypospadias repair surgeries, 13.3 % stoma creations, 11.5% anorectoplasties, 11.1% pull-through procedures, 6.2% thoracotomies and 2.7 % tube thoracostomies and of which 74.6% of patients received intra operative local or regional blocks. Among this 66.7% were caudal blocks, 6.2% were intercostal blocks and 1.8% were rectal sheath blocks (Table 1).

**Table 1:** The type of regional or local blocks given during the intra operative period in children under five years of age at Tikur Anbesa Specialized Teaching Hospital, Addis Ababa, Ethiopia

Type of blocks	Frequency	Percent
Not Given	57	25.3
Caudal Block	150	66.7
Intercostal Block	14	6.2
Rectal Sheath Block	4	1.8
Total	225	100.0

Among the 225 patients operated and had complete documentation, paracetamol (PCM) was the only analgesic medication ordered in 149(66.2%) of the cases. Ibuprofen and PCM were ordered in 73 (32.4%) patients and in the case of the remaining 3 (1.3%) patients. there was no properly documented order.151 (67.1%) patients were given PCM only, 72(32%) were given PCM and Ibuprofen and two patients didn't take any kind of analgesic in the postoperative period (Table 2).

**Table 2:** The type of post operatively ordered analgesia in children under five years of age at Tikur Anbesa Specialized Teaching Hospital, Addis Ababa, Ethiopia

Type of medication	Type of analgesic ordered	Type of analgesic given
Not ordered /not given	3 (1.3%)	2 (0.89%)
Paracetamol only	149 (66.2%)	151 (67.1%)
Paracetamol and Ibuprofen	73 (32.4%)	72(32%)
Total	225(100%)	225(100%)

**Figure 1:**
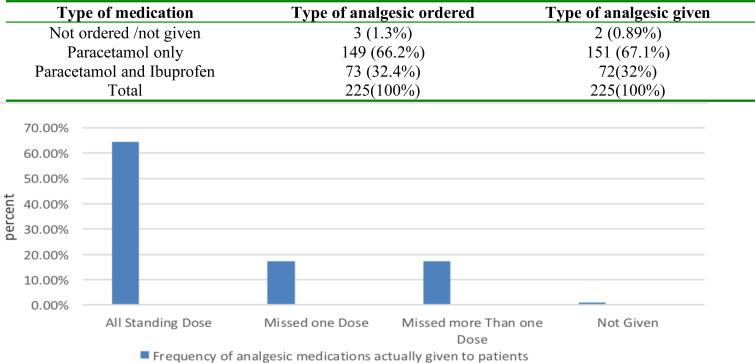
Frequency of post-operative analgesics actually given analgesia to children under five years of at Tikur Anbesa Specialized Teaching Hospital, Addis Ababa, Ethiopia

Regarding the frequency of order of analgesic Medications, in 98.67% ordered as standing dose but in three cases there was no order of analgesic medications at all and in the implementation of the orders only 64.4% of patients were given the analgesic medications on standing doses and also 34.6 % of the patients missed one or more doses. Two patients were not given any kind of analgesic medication in the first 48 hours post operatively (Figure 1).

**Figure 2:**
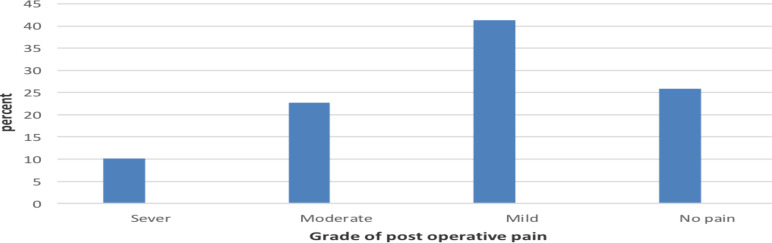
Grade of post-operative pain felt by children under five years of age who undergone major surgery at Tikur Anbesa Specialized Teaching Hospital, Addis Ababa, Ethiopia.

In respect to pain records and grade there was no any form of pain assessment mechanism in the wards or NICU but on subjective enquiry only 25.78% of patients didn't have any pain record during the assessment times. The remaining 74.2% had one or more records of pain. During the assessment of Grades of Pain felt by patients, it was found that there was no pain record in 25.78% of the patients. However, 74.2% of the patients had records of mild to severe pain in the first 48 hours after surgery (Figure 2).

Out of the 80 patients who missed one or more doses of post-operative analgesic medication, the reason for 46.25 % of the parents not to give the medication was confusing the drugs for antipyretics and refraining from administering them in the absence of fever. The other 37.5 % were not informed about the frequency and dose of administration or were not given prescriptions. 20% of the patients were not given the drugs because they were on their NPO period and parents thought oral analgesics ordered were contraindicated. 8.75 % of the parents didn't know how to administer the drugs. The percentage of patients who felt moderate to severe pain is higher in the groups who were taking paracetamol only compared to those who were taking paracetamol and ibuprofen together (Table 3).

**Table 3:** Type of analgesics given in relation to grade of the pain in children under five years of age who undergone major surgery at Tikur Anbesa Specialized Teaching Hospital, Addis Ababa, Ethiopia

Type of analgesic given	pain grade the patient felt				
	
	No Pain	Mild	Moderate	Sever	Total
Not given	0	0	0	2	2
Paracetamol only	34	60	40	17	151
Paracetamol and Ibuprofen	26	31	12	3	72
Total	60	91	52	22	225

Among 145 patients that were given all the standing doses of analgesic medication 52 patients felt pain once and 39 patients felt more than once. Among the 39 patients that missed one dose of the post-operative analgesic medication, 25 patients felt pain once and only one didn't have any pain record during the assessments. Among the 145 patients who were given all the standing doses of analgesic medications only 7 patients felt moderate to severe pain. 54 patients had no pain record and 51 of the remaining 84 patients felt mild pain. Out of the 39 patients who missed one dose of analgesic medication 33 patients had moderate to severe pain. Among the 39 patients who missed more than one dose of post op analgesic medication, 32 patients had moderate to severe pain record.

All patients who underwent thoracotomy and 83.3 % of those who underwent tube thoracostomy had non-zero records of pain.78.57 % of the former and 66.67 % of the later groups had high grade pain records in the first 48 hours after their procedure. 51% of the patients who underwent pull though surgeries ad 38.4 % of those who underwent anorectoplasties,18.18% of stoma creations and 26.08 % of stoma reversals had high grade pain records.

## Discussion

There were four main findings in this study. First, there was no pain assessment mechanism in the wards and NICUs for post-operative paediatric patients. Second, higher number of patients missed one or more doses of analgesic medications ordered in their post- operative period. Third, significant number of patients who were not given all the doses of analgesic medications had higher grades of pain (moderate to severe) in their post-operative period. Fourth, majority of patients that had records of moderate and severe pain were taking a single modality of pain management in the post-operative period. Pain assessment records, including the characteristics of pain and the patient's analgesic response, guide the medical team for providing effective pain management. In this study, there was no any pain assessment done by ward and NICU nurses. There was no pain assessment tool attached in the charts of the patients. There was no documentation of analgesic medications given to patients on nurses' medication sheet. This study is comparable to a study done in Turkey on postoperative pain assessment records of nurses which showed that there was no pain assessment in any of the patient files with in the first 48 h (10). Another study in Finland showed that although nurses have several valid and reliable tools at hand for the evaluation of pain in children, they do not generally use them. Only one third of the nurses knew about existing pain measurement instruments in their wards (11). In this study 78(35.56%) patients have missed one or more doses analgesic medications in the first 48 hours post operatively and two patients missed all the doses. This result is comparable to a study done in Kenya which showed that Pain medicine had been prescribed to 66% of participants at some point during their hospitalization. Only 35% of subjects received their pain medicines as prescribed within 72 hours. Frequent and prolonged stock-outs of pain medications, staff shortages, system failures and under prescription by clinicians were some of the reasons mentioned in Kenyan study for improper administration of pain medications (12). Although the reasons mentioned above could not be totally ruled out by this study, the most common reason for missing doses is poor understanding of parents about the proper administration of the analgesic drugs. In this study 32.89 % of patients felt moderate to severe pain in the first 48 hours post operatively. This number is comparable to studies done in Kenya and Turkey. A study done in Kenya showed that out of 400 hospitalized patients, ranging in age from 5 to 88 years old, 49% of patients had mild pain and 32% of patients had moderate to severe pain (12). Another study done in Turkey showed 36% of 815 analyzed children and adolescents suffered from clinically significant pain during their entire hospital stay (13).

In conclusion, though the number of patients with positive records of post-operative pain is higher, there is no pain assessment mechanism in the wards and NICUs for post-operative patients and significant number of patients missed one or more doses of analgesic medications in their post-operative period also positive records of pain were observed in patients who missed one or more doses of post-operative analgesics. Despite the number of patients with mild and moderate pain records most took only one type of analgesic medication. The knowledge of parents or legal guardians towards the proper administration of analgesic medications is limited making neonates and under-five children to suffer from post-operative pain. Thus, post-operative Pain assessment in children should be conducted on regular bases with assessment tools. Educating and supervising care givers on proper administrations of drugs should be routine with strict application of multimodal pain management protocols based on grade of pain and availability of drugs in the post-operative periods. We recommend further study to address the treatment outcome and adverse effects of the medications.
